# Global DNA Methylation in the Limbic System of Cattle

**DOI:** 10.3390/epigenomes3020008

**Published:** 2019-05-05

**Authors:** Bonnie Cantrell, Hannah Lachance, Brenda Murdoch, Julia Sjoquist, Richard Funston, Robert Weaber, Stephanie McKay

**Affiliations:** 1 Department of Animal and Veterinary Sciences, University of Vermont, Burlington, VT 05405, USA; 2 Department of Animal and Veterinary Science, University of Idaho, Moscow, ID 83844, USA; 3 Department of Neurological Sciences, University of Vermont, Burlington, VT 05405, USA; 4 West Central Research and Extension Center, University of Nebraska, North Platte, NE 69101, USA; 5 Department of Animal Sciences and Industry, Kansas State University, Manhattan, KS 66506, USA

**Keywords:** epigenetics, methylation, cattle, temperament, brain

## Abstract

To elucidate the extent to which DNA methylation varies across multiple tissues in the brain and between animals, we have quantified global DNA methylation in tissues comprising the limbic system for six Red Angus x Simmental steers. Global DNA methylation was measured in nine regions of the bovine brain: amygdala, the bed nucleus of the stria terminalis, cingulate gyrus, dorsal raphe, hippocampus, hypothalamus, nucleus accumbens, periaqueductal gray and prefrontal cortex. DNA methylation varies among animals for each tissue type and varies among tissue types for each animal. The highest amounts of DNA methylation were found in the amygdala, cingulate gyrus and dorsal raphe, while the bed nucleus of the stria terminalis, nucleus accumbens and periaqueductal gray had the lowest amounts of DNA methylation. A heatmap sorted by *k*-means clustering was generated to graphically display percent DNA methylation in relation to tissue type and animal number. This is the first study to report measures of DNA methylation in the limbic system of the bovine brain and can be used to inform the cattle genomics community of expected variation in cattle brain methylation.

## 1. Introduction

Epigenetic modifications are a source of phenotypic variation that are independent of DNA sequence variation. Epigenetic modifications do not change the DNA sequence itself but can affect transcription and are important mediators of gene expression [1]. Consequently, epigenetic modifications can cause one genotype to produce alternative phenotypes. DNA methylation is a type of epigenetic modification that in mammals primarily involves the addition of a methyl group to the 5 position of cytosines that are found in CpG dinucleotides. When 5-methylcytosine (5-mC) is found in the 5′ promoter region of a gene, it can prevent transcriptional machinery from binding to its target, resulting in the silencing of gene transcription [2].

DNA methylation patterns are unique in mammalian brains. Brain functions impacted by DNA methylation include motivational and emotional behavior, which are regulated by the limbic system [3]. The limbic system has been investigated primarily in rats and mice for variation in global DNA methylation with regard to addiction, nutrition, disease, stress, and aging [4,5,6,7,8,9,10,11,12,13,14]. Gibbs et al. [15] quantified DNA methylation levels for the human cerebellum, frontal cortex, temporal cortex, and pons. Generally, methylation levels were similar between cortical brain regions and the brainstem, but different from the cerebellum. These studies highlight the importance of examining multiple tissues within the brain to determine the extent of epigenetic modulation. 

Comprehending the role of DNA methylation relative to function and complex traits requires knowledge of the global representation of tissue specificity and variation. Our study focuses on nine tissue types within the limbic system, of which three have never been examined for global DNA methylation in any species. Here, we have quantified the variation of global DNA methylation within the limbic system of cattle. 

## 2. Results

**Figure 1:**
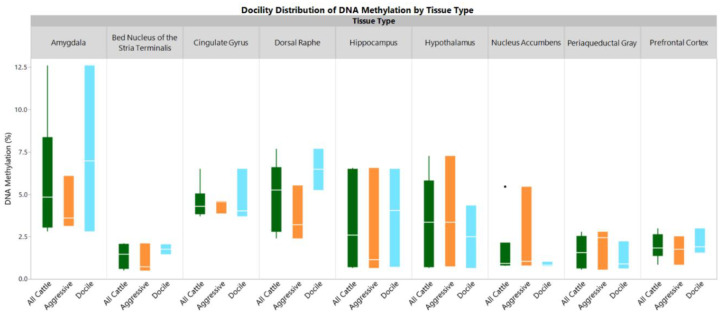
Distribution of Global DNA Methylation by Tissue Type.

**Figure 2:**
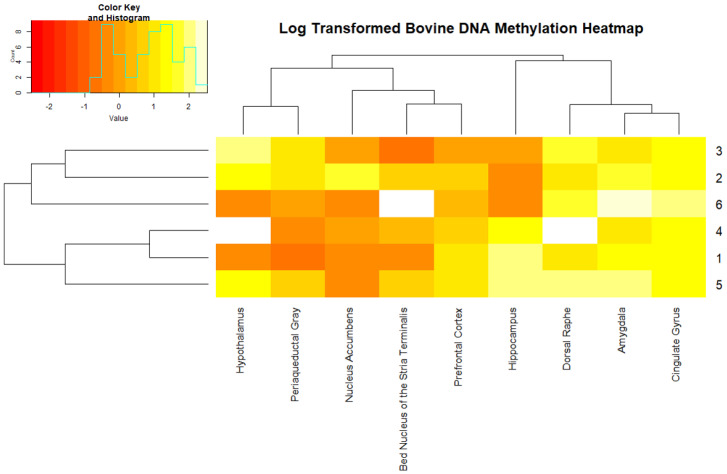
*K-*means Sorted Heatmap of Global DNA Methylation by Tissue Type and Animal Number.

Average measures of global DNA methylation ranged from 1.38 ± 0.74% in the bed nucleus of the stria terminalis to 5.87 ± 3.70% in amygdala (Figure 1). DNA methylation levels were heterogeneous within tissue types and within each animal (Figure 2). The dorsal raphe, amygdala, and cingulate gyrus contained consistently higher amounts of DNA methylation in each animal. Conversely, the lowest amount of DNA methylation in most animals was found in the nucleus accumbens. The exception was animal 2 that showed a higher level of methylation in the nucleus accumbens but was the outlier in the boxplot (Figure 1). 

Outlier box plots are shown for each tissue type based on the percent of methylation. The solid box outlines the 1st and 3rd quartiles with a line in the middle representing the median. Whiskers extend up to the distance of the interquartile ranges. The ends of the whiskers are the highest or lowest percent of methylation within a normal distribution. A black dot indicates an outlier in the distribution. 

A heatmap of the log-transformed DNA methylation levels for nine tissues of the bovine brain in six animals. The key identifies the log-transformed DNA methylation percentage as “Value” and the number of animals for each methylation percentage is labeled as count. Tissues and animal numbers are sorted according to *k*-means clustering results. Three samples have missing data indicated by a white box (animal 4’s dorsal raphe and hypothalamus and animal 6’s bed nucleus of the stria terminalis). 

Discrepancies between hierarchical clustering and *k*-means clustering were identified when examining DNA methylation levels. Clustering tissue type with three centers for *k-*means generated three clusters: (1) amygdala, cingulate gyrus, and dorsal raphe, (2) the bed nucleus of the stria terminalis, hypothalamus, nucleus accumbens and periaqueductal gray, (3) hippocampus and prefrontal cortex. Hierarchical clustering identified three clusters: (1) amygdala and cingulate gyrus, (2) the bed nucleus of the stria terminalis and prefrontal cortex, (3) hypothalamus and periaqueductal gray (Figure 2). For both methods, the amygdala clustered with cingulate gyrus and hypothalamus clustered with periaqueductal gray, thus, indicating more similarity in DNA methylation levels in these two pairs of tissues compared to other tissues measured. 

## 3. Discussion

Each tissue within the brain, although interconnected through neural pathways, has its own unique functions that may be contributing to differences in DNA methylation between each tissue type. The periaqueductal gray has been shown to be connected to the hypothalamus through the oxytocinergic social network [16]. We found DNA methylation levels to be similar between the hypothalamus and periaqueductal gray (Figure 2), which may indicate a similar use of DNA methylation for the oxytocinergic social network. We also found the amygdala and cingulate gyrus cluster consistently together, which may indicate similar epigenetic regulation between these tissues. The amygdala, cingulate gyrus and dorsal raphe have the highest average and nucleus accumbens has the lowest average percentages of global DNA methylation (Figure 1 and Supplementary Table S1). Cingulate gyrus and dorsal raphe are important intermediates in neural signaling pathways of neurotransmitters [16,17]. The cingulate gyrus is an integration point of the emotion and memory processing limbic cortex and the decision-making cerebral cortex. The amygdala is important for the regulation of signaling within the limbic system involving the signaling pathways of serotonin, glutamate, and dopamine and is thought to be the main regulator of the hypothalamus’s function [17]. These tissues may utilize methylation to regulate signaling processing differently for each pathway that it interacts with, thus partially explaining the higher percentages of global DNA methylation. Interestingly, the hippocampus showed the greatest difference between animals with respect to log-transformed DNA methylation level and clustered animals into two groups (Figure 2). This elucidates the potential of global DNA methylation in the hippocampus to play drastically different roles within cattle. 

Global DNA methylation levels and variation across samples within this study are similar to other studies. Global DNA methylation levels less than 1% are not uncommon for brain tissues. Reports of global DNA methylation in the hippocampus have ranged from >0.01% to 1.15% across rat studies using the same global quantification kit as the current study [18,19]. We found that half of our animals had global DNA methylation levels less than 1.15% in the hippocampus. However, the other half of the animals had values greater than 4%. Similarly, we found that two of the five animals dissected for the hypothalamus have global DNA methylation levels within the previously reported range of 0.5% to 1.25% and the remaining samples have greater than 3.3% global DNA methylation [20]. While these levels of global DNA methylation seem high for brain tissues, our data are similar to that previously reported for cattle [21]. In 2018, Mendonça and colleagues quantified global DNA methylation in blood, funiculus umbilicalis, cotyledon, and allantochorion tissues in newborn calves using the same MethylFlash kit (Epigentek, Farmingdale, NY, USA) as the present study. Mendonça reported high levels of global DNA methylation across tissues (10.71–24.13%) in male calves and a standard error of mean up to 4.31% in males and 7.56% in females [21]. We found DNA methylation to range from <1% to 12.5% across all tissues and a variation across animals between 2% and 10% within a single tissue. This variation is higher than the previous cattle study but could be a result of tissue-specific variation across animals.

The extent of knowledge pertaining to DNA methylation in brains of livestock species is severely lacking. In order to elucidate the relationship between methylation and economically important traits and diseases in brains, we must first establish a baseline for global DNA methylation, which has been reported here. To the best of our knowledge, this is the first examination of DNA methylation in the limbic system of cattle and the first study to examine global DNA methylation in the bed nucleus of the stria terminalis, cingulate gyrus and dorsal raphe in any species. With a renewed interest in the increased understanding of the brain and its functions, the focus on behavioral and nutritional epigenetics and the intricacies of the cattle brain epigenome have never been more important.

## 4. Materials and Methods

### 4.1. Animals and Tissue Collection

The University of Nebraska Animal Care and Use Committee approved (IACUC #1202, approved on 1 August 2013) the procedures and facilities used in this experiment. Brains from steers crossed with 5/8 Red Angus and 3/8 Simmental were collected at slaughter (Tyson Foods, Lexington, NE, USA), and shipped on dry ice overnight to the University of Vermont. Upon arrival, brains were double bagged in 8-mm-thick zipper bags and stored at −80 °C until dissection. The following tissues were dissected: amygdala, cingulate gyrus, hippocampus, nucleus accumbens, periaqueductal gray and prefrontal cortex (*n* = 6 each) and the bed nucleus of the stria terminalis, dorsal raphe and hypothalamus (*n* = 5 each). All steers were less than 20 months old at time of slaughter, were fed the same diet, and were housed at the same facility.

### 4.2. Sample Processing

For each of the functionally distinct tissues of the brain that were dissected, DNA was extracted using a modified version of the DNA Extraction Kit’s Protocol II: Extraction from Whole Tissue from Agilent Technologies (Santa Clara, CA, USA). Briefly, all solutions used were scaled down for the use of 9 mg of each tissue. Samples were incubated at 56 °C for 14 h with shaking using a Shake ‘*n*’ Bake Shaking Hybridizing Incubator (Boekel Scientific, Feasterville, PA, USA). Once 100% ethanol was added, samples were spun at 2000× *g* for 15 min. The supernatant was discarded and the DNA pellet was washed with 70% ethanol and spun at 2000× *g* for 5 min. The supernatant was again discarded and the pellet was left to air dry for an hour to overnight. DNA was resuspended in 10 mM Tris and 0.1 mM EDTA buffer. It is important to note that this DNA extraction method does include both RNase and protease steps to assist with eliminating non-DNA material. The DNA was quality checked for contamination using the NanoDrop 1000 Spectrophotometer (Thermo Scientific, Wilmington, DE, USA) and quality checked for the absence of DNA sheering using a 1% agarose gel with ethidium bromide.

### 4.3. Global DNA Methylation Measurement

All samples were tested for global DNA methylation using the MethylFlash™ Methylated DNA Quantification Kit (Colorimetric) (Epigentek, Farmingdale, NY, USA). The MethylFlash (previously called Methlamp) kit is advertised as having an accuracy of 99% in determining global DNA methylation [22]. MethylFlash uses an enzyme-linked immunosorbent assay (ELISA) based method to quantify global DNA methylation. This method of global DNA methylation quantification has been adopted by multiple antibody and epigenetic companies including Zymo (Irvine, CA, USA), Abcam (Cambridge, MA, USA), Sigma (St. Louis, MO, USA), and Cell Bio Labs (San Diego, CA, USA). However, of these methods, Epigentek’s MethylFlash kit was published more frequently [23]. Studies that have compared immunoquantification and ELISA-based assays of global DNA quantification have found the results to be comparable to other methods of global DNA methylation, but there is a greater limitation on detection range of DNA methylation and robustness in ELISA based methods [23,24,25]. ELISA-based methods are easier to use and cost less than mass spectrometry and chromatography-based methods, allowing for ELISAs to be more applicable in clinical settings [25]. The standard protocol was modified to better control for the variation between duplicates on the plate as the accuracy of ELISA-based kits can be limited if washing and the various antibodies timing are not first optimized [26]. 

For each sample, 50–200 ng of DNA was used, in duplicate, as recommended by Epigentek. Epigentek states that the MethylFlash kit specificity binds single- or double-stranded DNA 100 bp or larger to the assay plate. The manufacturer’s standard absolute quantification protocol was modified according to recommendations from Epigentek (personal communication). Briefly, those modifications were the use of a multichannel pipette, use of one set of tips to add a solution and a new set for removal of solution, avoiding contact with the sides of the wells of the plate, tilting the plate to remove solutions, keeping consistent timing during color changing steps, and avoiding exposure to light during the color changing steps. In order to decrease the percent coefficient of variation between duplicate samples, additional modifications were implemented for seventeen samples. Specifically, to remove solutions, the plate was inverted quickly and hit on an absorbent pad until the wells appeared dry. Absorbance readings from each plate were calculated at 450 nm using a BioTek Synergy Plate Reader and the Gen 5 Program Data Analysis Software V.2.03 (Winooski, VT, USA). 

### 4.4. Data Analysis

A standard curve was generated in Microsoft Excel 2013 (Redmond, WA, USA) using control samples (provided in the kit) from each plate. Subsequently, the percent coefficient of variation (CV) was calculated for the optical density (OD) values of the duplicates:

$$\frac{Standard\;Deviation}{AverageOD}\times 100=\%CV$$

Samples with a less than 15% CV were considered passing for each plate as recommended by Epigentek (personal communication). Percent global DNA methylation was calculated using Epigentek’s equation for absolute quantification, which accounts for the amount of DNA used for each sample. 

Descriptive statistics were generated in RStudio version 1.0.136 (RStudio, Boston, MA) and JMP^®^ Pro 12.1.0 (SAS Institute Inc., Cary, NC, USA). Outlier box plots were constructed in JMP^®^. The distribution of DNA methylation was skewed to the right and therefore a log transformation was performed to normalize the percentages of DNA methylation. The heatmap and *k*-means were generated in RStudio for log-transformed data. Heatmaps were created using heatmaps.2 within gplots 3.0.1 sorted for tissue type and animal number by k-mean clustering with no trace [27]. The heatmaps.2 algorithm creates a dendrogram based on agglomerative hierarchical clustering. This method builds a distance matrix starting with one node for each sample and repeating until there is one node for all samples. The Lance–Williams dissimilarity formula is used by the program to cluster the samples by Euclidean distance. There were missing data for the bed nucleus of the stria terminalis, dorsal raphe, and hypothalamus and therefore *k*-means clustering used the k-POD algorithm for a maximum of 100 iterations in the kpodclustr package to account for the missing data [28]. The *k*-means clustering method starts with a random sample as its center of the cluster for a *k* number of centers. Similar to hierarchical clustering, the Euclidean distance is calculated between samples to the nearest center. However, the mean distance to the center of all samples in the cluster is used to calculate the location of the next center and the calculation is repeated. The *K*-POD clustering results were based on two centers for comparison between animals and three centers for comparison between tissue types. Biologically, it was unknown how different tissues would cluster in respect to DNA methylation levels and therefore to determine the number of centers, the RStudio package ‘stats’ (version 3.2.5) hclust function was used to cluster by Euclidean distance with the ward.2 cluster method. The number of centers used for tissue comparisons were based on the tightness of clustering in the output of Ward’s hierarchical clustering.

## Supplementary Materials

The following are available online at https://www.mdpi.com/2075-4655/3/2/8/s1. Table S1: Table of Percent Global DNA Methylation.
